# Mosaic and Modern Dietary Laws

**Published:** 1885-02

**Authors:** H. Lassing


### BUFFALO

### Medical and Surgical Journal.

Vol. XXIV. FEBRUARY, 1885. No. 7.

### Original Communications.

### Mosaic and Modern Dietary Laws.

By H. Lassing, M. D.

A writer in a foreign magazine accounts for the longevity of the Israelite by ascribing it to the observance of the Mosaic and Rabbinical laws relative to marriage, the relations of the sexes, the hygiene of married life, and their strict dietary regulations. These latter were evidently designed for the maintenance of their health and strength and the protection of their bodies against disease. Thus we find included among the prohibited sources of food all carnivorous animals, the rodents, the carnivorous and carrion eating birds, reptiles, amphibia and mollusca, a list comprising a complete group of beasts, such as the swine, the mouse, the rat, the cat and the dog, etc., known to be perfect foci of trichinal and other parasites. The communicability to man of parasitic diseases from animals used as food has long been placed beyond all doubt, it having been established that the parasite is simply transferred from the flesh of the beast to that of the man, in which it develops with frequent fatal results. The explanation of the reason for the prohibition of scaleless fish, such as the eel, only recently made by the scientists bears out this theory. It is that, owing to the absence of scales, the eel becomes a positive absorbent of noxious gases, more particularly the noxious effluvia of decomposing, and, therefore, poisonous matter.

These dietary laws are not confined to a mere division of all animals into two classes, the "clean and unclean." They prescribe even how much of the bodies of permitted animals may be consumed as food. Thus the use of blood is emphatically and repeatedly forbidden. This prohibition and the importance evidently attached to it harmonize so exactly with the lessons of modern science that it is impossible to regard them as motived by any consideration other than the public health.

The possibility of the blood containing disease germs, not immediately affecting the quality of the flesh, is not the only circumstance tending to disqualify it for food.

The blood, in its normal condition, almost invariably contains noxious elements. From the very nature of the double office of the circulatory system this must be so; for while, on the one hand, the blood serves to renew the various parts of the system after their ordinary wear and tear, on the other, it has to carry off the natural waste of the tissues. This waste or refuse is ultimately eliminated by means of the kidneys, the sudoriparous glands, etc., and then appears in its avowed character of excrementitious matter; but it must always be, to a certain extent, present in the blood, and, in the event of any derangement of the action of the kidneys, accumulates in considerable quantities and highly poisonous qualities. It must, therefore, be evident that the blood is always an undesirable article of food, especially as it is impossible, when an animal is slaughtered, to separate the arterial from the venous blood, which would be the only means of overcoming the difficulty.

"We contend," says one writer, "that to use the blood as food approximates very closely to drinking urine, and is not merely loathsome, but, *pro tanto*, unsafe. That, like liquid and solid excrement, it is valuable for plant food, and that it serves as pabulum for certain classes of animals, is no proof that it is fit for human consumption."

The old Jewish Rabbins well knew the importance of this, some seventeen hundred years ago, when they recommended that in slaughtering animals, in addition to severing the trachea and œsophagus, the blood should be poured out from the vessels of the neck, and this at a time when it was believed the arteries contained only air.

One of the most important features of the Jewish system was an elaborate examination of the carcass before it could be declared fit for Jewish food; this examination, prescribed by the Rabbins and faithfully carried out at the present day, is of an extremely vigorous and subtle nature, and completes the system by which the selection of animal food is governed. The conditions on which alone the flesh of animals is permitted to be eaten are singularly minute, and these laws, carried out in their integrity, render the consumption of meat affected with specific maladies practically impossible. The lung is especially ordered to be examined and tested so that pleuro-pneumonia, tuberculosis, bronchitis, and pulmonary maladies generally, have little chance of escaping detection. "The extreme care of these early students of physiology (the Rabbins)," says Dr. Maurice Davis, "in their examination of the lungs, seems to point to the dicta of modern science, which indicate the air passages, with their moist mucous membranes, as highly probable inlets of the morbid particles floating in the atmosphere." Dr. Behrend tells us that the animal diseases transmissible to man through ingested meat are seven in number, viz., cattle plague, swine typhoid, pleuro-pneumonia, foot and mouth diseases, anthracoid diseases, erysipelas and tuberculosis. By the observance of Jewish dietary laws it is impossible for animals affected by any of these diseases to be eaten. On the other hand, under non-Jewish systems these diseases are spread broadcast with criminal recklessness. Dr. Carpenter stated, some time ago, in the *British Medical Journal*, that an inspector of the Metropolitan meat market, in London, had declared upon oath that eighty per cent. of the meat sent to the London market had tubercular disease; and a letter addressed by a Mr. Jenkins to the *Times* a few months ago, calculated in reference, to this same fact, that "at least 375,000 of the inhabitants (of London) annually run the risk of being tainted with consumption and of transmitting it to their unborn children." What wonder then that tuberculosis has so many victims?

One of the most important considerations for the physician is the quality and character of the diet of his patient, as well as of the community in which he lives. The most valued of articles of human food is beef. It was a favorite aphorism of one of our learned professors that beefsteak is the best tonic. This, to a very large extent, is true, and, therefore, all other things being equal, it must be of vital interest to the physician to have the supply of beef of the very best, and to have it in that condition which will furnish the greatest amount of healthy nutrition in the most suitable form, palatable, and at a comparatively low price.

To speak on this matter intelligently, it is necessary, first, to consider what are the characteristics of good beef, what should be avoided and what required in its production.

Good beef, then, should be bright red in appearance, the muscular parts firm and elastic, the fat hard and light, yellow or white, it should be free from all substances calculated to expedite putrefaction, such as exuded blood, bruises or extraneous dirt. To produce such beef it follows that the animal from which it is taken must be in a perfectly healthy condition and of the proper age when slaughtered, and that the process of slaughtering and preparing for market must be conducted in such a way as to obviate anything tending to vary from the conditions just enumerated.

Certain conditions may be enumerated which invariably tend to produce poor, unhealthy, as well as unsavory beef, while others, just as surely, will result in good, healthy and appetizing meat. No one can deny that long driving and exhausting railroad journeys in crowded cattle cars, with insufficient food and water, the animals goaded, beaten and bruised by brutal drivers, frightened by unusual noises, will cause inflammatory, congested condition of the meat and can never result in good wholesome beef. Again, the careless handling of the dressed meat, filth and dirt allowed to accumulate about the meat in the slaughterhouse, insufficient emptying of the blood from the carcass, all injure the quality of the beef.

And yet another consideration, which is, that beef should be long enough killed before it is cooked to have allowed of complete relaxation of the muscular contraction of the fibre without permitting even the slightest decomposition to take place. We must not for a moment lose sight of the fact that any process of decomposition may be arrested by antiseptics, yet can never be undone, or in other words, the destructive work can never be so remedied as if it had not taken place.

It is clear, then, that healthy animals of a proper age, well rested, fed and watered, killed in such a manner as to produce the least fright and congestion, dressed in the cleanest possible manner, completely deprived of the excessive blood and animal heat, and so kept as to prevent even the slightest decomposition, will furnish the best, most wholesome and appetizing beef.

The last consideration is the availability of such good beef to the largest number of people. The nearer to the point of production and the more direct a product is brought from the producer, the cheaper it must be.

Having thus spoken of the characteristic requisites and cost of wholesome, well-tasting beef, we will now briefly consider if such can be so found within our reach.

In Chicago, one thousand miles nearer the vast cattle producing pastures of the far West, are the largest stock yards in the world. Connected with the entire Union by the most extensive system of railways centering here, cattle are brought here from all directions, but more largely from the vast ranches of

Montana, Colorado, Nebraska and Texas, and after being fully rested, fed and watered, are picked out and bought by a vast army of buyers for local use as well as for re-shipment. The principal grades of cattle here represented may be divided for our purpose into home raised and range cattle, and it may be stated that the quality of these latter has been vastly improved during the last few years, not only by a more careful breeding, but principally from the fact that so many railroads are now operated through the country that it no longer pays to drive these range cattle on foot only for the shortest distances to the nearest railway station.

The cattle, having thus been carefully selected and purchased, are driven to the cattle-pens of the different slaughter and packing houses which surround the stock yards and form a very large suburb of Chicago.

To give a correct idea of what seems the best method of slaughtering and dressing animals for the supply of wholesome, good beef for the Eastern markets, we will now enter one of these establishments and follow a steer from the pen to the market in Boston, New York, Liverpool or London.

One of the largest of these beef dressing places, located on the edge of the stock yards, is that of the Swift Brothers, whose name on refrigerator cars and large refrigerators in the Eastern markets has become so familiar.

The other is that of Armour & Co. These are the two firms who are what are known as Chicago beef dressers. It may be added here that beef dressing simply means the preparation of beef after killing, by cleansing it of all uneatable debris and offal and not, as some intelligent people even have imagined, the application to the meat of any preservative mixture or wash.

The bullock to be killed is driven from the enclosure wherein he is confined to a separate pen and thence into a narrow stall where he is alone by himself. Here he is stunned by one or two blows upon the head, which has a double object, one to disable him, but the more important object is to drive all the blood possible to the brain and forward parts. After this a chain is fastened to him and through a sliding door he is hauled upon what is called the bed of the slaughter house. Overhead throughout the entire buildings, is a system of iron tracks, intersecting, and connected by switches, to which are attached sheaves and blocks, with hooks, and these, being connected with powerful steam machinery, may be run in any direction. After the steer is thus placed upon the bed, his fore feet are so placed as to open as wide as possible the opening which is to be made in his arteries, and thus make sure of the perfect and entire exit of blood. He is again knocked in the forehead until his muscles relax, when a man opens, with a sharp knife, one of the largest arteries in the body at the lower throat; his head is then entirely severed from the body, and, by means of a chain on the hind legs, he is hoisted up clear of the floor, and shoved into another place to make room for a fresh victim; he is again lowered on the floor, where he is placed on his back, his feet taken off, the skin taken off the belly and sides, the breast and crotch sawed open and the caul taken off. This caul, surrounding the stomach, contains the purest fat in the steer, and is washed and cleansed to be converted into the finest and most valuable suet oil. At this stage the examination of the lungs is made, and if any evidence of disease is found the carcass is prepared for other uses than refrigerating, as it has already been shown that such meat will not keep. Thus, in the self-interest of these Chicago beef dressers, lies the greatest safety of the consumers of this kind of beef. The carcass is then wiped all over, and washed free from all blood stains and other impurities; he is then hooked and hoisted up part way, the tail is cut out, furnishing ample material for the celebrated ox-tail soup, the rump is skinned, and, after being hoisted clear of the floor, two men beat down the fell on the hind legs, another takes the hide off the back, the entrails are removed and the animal is sawn down the spine from the tail, the back is thoroughly washed off and wiped and split down the middle; he is then hoisted further and run on the track to more thoroughly drain out the blood, the hide is entirely cut off from forelegs and neck, the last attachment, and this is done in such a neat, careful way, with clean knives and hands, that the hides from this establishment bring an extra price in the market. The neck is split and the carcass is trimmed carefully, cutting off all ragged pieces of fat or meat, all blood stains or bruises. It is again thoroughly washed in two waters and wiped dry, when it is allowed to hang at the back of the beds near the cooler, where, after being allowed to thoroughly drip some fifteen minutes or more, it is again wiped and trimmed and shoved in the cooler to be entirely deprived of its remaining animal heat. It will thus be seen that the carcass is not only kept very clean, thoroughly emptied of blood, being about one hour or more in transit from the pen to the cooler, but none of the meat is ever, under any circumstances, allowed to touch the floor. The number of men actually handling the animal from the pen to the cooler is forty-two. In addition to these there are large numbers carrying away offal, washing floors and posts, wiping the hooks and implements, so that at the close of each day's killing there is not any filthy debris or even blood left on the floor. While it is unavoidable for those who do the bloodiest work to keep from soiling their hands and clothing, they are, by the strictest discipline, compelled constantly to wash their bodies, and the clothing is changed and washed at every resting spell. All debris is instantly removed and worked up to some useful purpose, so that there can be nothing remaining to cause any foul smell or decomposition, and in the various buildings on the grounds, though every process of manufacture of the debris is carried on, no smell of an objectionable nature is perceptible, and cleanliness is carried to such an extent that a lady with kid gloves could fearlessly handle the woodwork of the coolers and the meat therein contained without soil or stain. The floors of these coolers are covered with clean, dry sawdust, which is changed every other day. These coolers or refrigerators are very simple in construction, with hollow walls having an ice chamber overhead, the cold air from which is conducted downward, and thus is kept up a constant circulation of pure air at an even temperature of about 40° F. These refrigerators have ample room to hold about 7,000 cattle. The cars in which this beef is transported are built on a similar principle, and will each contain from twenty-five to thirty-four cattle. The meat is usually kept forty-three hours before shipment, by which time all animal heat is surely expelled from the meat. It is from three to four days on the way to Boston, New York, Philadelphia or Baltimore, and on the way the ice is re-supplied four times. Thus beef sent from this place is at least seven days old when it reaches the New York market. In the winter time the same temperature is maintained, but of course the ice needs only to be replenished twice.

Beef which has been thus refrigerated will keep out of the refrigerators perfectly sweet and wholesome two days, even in the hottest weather. It is unnecessary to state that ordinary beef, not so well cleansed and not having the animal heat so thoroughly removed will not keep nearly so long.

This concern uses about thirty-three car loads of fresh ice per day. As each car averages fourteen tons, this makes about 500 tons of ice per day.

Ice is very cheap here, so much so that it does not even pay to manufacture artificial ice, much less to use any chemical or other artificial means of cooling.

One notable fact connected with this process of slaughtering cattle is that the hearts of animals so killed are always empty of blood, whereas, those killed by other processes are generally full. Beef thus prepared is sent by rail to the coast, shipped in similar refrigerators on steamers to Liverpool and London, where it is taken out of the coolers, carted and sent by ordinary rail car to Smithfield and other markets in England and has always been in good condition and superior to English beef. In one instance meat was thus kept forty-three days on a disabled steamer and yet was in fair condition on arrival in England.

There has been considerable prejudice created against this so-called Chicago dressed beef by ignorant and malignant misrepresentation, sometimes willfully confounding it with inferior canned beef, which, because it is also packed in Chicago, has been called "Chicago beef"; and, again, the writer has frequently seen butchers selling Chicago dressed beef as home dressed beef, while they were vigorously declaiming against this Chicago stuff and pointing out inferior home beef as being Chicago meat. It has got to be quite customary now, with careless reporters, whenever, justly or unjustly, meat has to be spoken of as the canse of sickness, glibly to describe it as Chicago beef, thus creating an unfounded prejudice against what is really a wholesome, palatable and cheap article of diet. Many districts of country which have heretofore suffered for the want of tender, toothsome beef, are now well supplied from this source. (About four thousand head of cattle are daily slaughtered in these establishments.)

Some of the prejudice against this beef has been created by the ignorance of the so-called leaders of working men, who led them to believe that by using Chicago meat they were injuring their fellow-workmen along the various railroads between Chicago and the Atlantic board, as well as those in the Eastern slaughter houses. The fallacy of this belief is too evident to require argument.

